# Inhibition of MMP8 effectively alleviates manic-like behavior and reduces neuroinflammation by modulating astrocytic CEBPD

**DOI:** 10.1186/s12974-024-03054-2

**Published:** 2024-02-28

**Authors:** Tzu-Yun Wang, Eddie Feng-Ju Weng, Yun-Chen Hsu, Lu-Ping Shiu, Teng-Wei Huang, Hsuan-Cheng Wu, Jau-Shyong Hong, Shao-Ming Wang

**Affiliations:** 1 Department of Psychiatry, National Cheng Kung University Hospital, College of Medicine, National Cheng Kung University, Tainan, Taiwan; 2 Neuroscience and Brain Disease Center, China Medical University, Taichung, Taiwan; 3 Graduate Institute of Biomedical Sciences, College of Medicine, China Medical University, Taichung, 404333 Taiwan; 4 School of Medicine, College of Medicine, China Medical University, Taichung, Taiwan; 5 Ph.D. Program for Aging, China Medical University, Taichung, Taiwan; 6 Neurobiology Laboratory, National Institute of Environmental Health Sciences, National Institutes of Health, 111 T.W. Alexander Dr., Research Triangle Park, Durham, NC 27709 USA

**Keywords:** Neuroinflammation, Bipolar disease, Ouabain-induced manic-like behavior, CEBPD, MMP8

## Abstract

There is an intrinsic relationship between psychiatric disorders and neuroinflammation, including bipolar disorder. Ouabain, an inhibitor of Na^+^/K^+^-ATPase, has been implicated in the mouse model with manic-like behavior. However, the molecular mechanisms linking neuroinflammation and manic-like behavior require further investigation. CCAAT/Enhancer-Binding Protein Delta (CEBPD) is an inflammatory transcription factor that contributes to neurological disease progression. In this study, we demonstrated that the expression of CEBPD in astrocytes was increased in ouabain-treated mice. Furthermore, we observed an increase in the expression and transcript levels of CEBPD in human primary astrocytes following ouabain treatment. Transcriptome analysis revealed high MMP8 expression in human primary astrocytes following CEBPD overexpression and ouabain treatment. We confirmed that MMP8 is a CEBPD-regulated gene that mediates ouabain-induced neuroinflammation. In our animal model, treatment of ouabain-injected mice with M8I (an inhibitor of MMP8) resulted in the inhibition of manic-like behavior compared to ouabain-injected mice that were not treated with M8I. Additionally, the reduction in the activation of astrocytes and microglia was observed, particularly in the hippocampal CA1 region. Excessive reactive oxygen species formation was observed in ouabain-injected mice, and treating these mice with M8I resulted in the reduction of oxidative stress, as indicated by nitrotyrosine staining. These findings suggest that MMP8 inhibitors may serve as therapeutic agents in mitigating manic symptoms in bipolar disorder.

## Introduction

Glial cell activation and oxidative stress have been reported to contribute significantly to neuroinflammation [1–3]. Bipolar disorder is a chronic mental disorder characterized by a range of mood symptoms, including mania, depression, and cognitive impairment, and causes substantial illness-related disability and mortality [4–6]. Although the underlying pathophysiological pathways of bipolar disorder remain unknown, neuroinflammation may be associated with bipolar disorder [7]. Na^+^/K^+^-ATPase, a transmembrane protein expressed in nearly all brain cells, is responsible for ATP-dependent, coupled transport of sodium and potassium ions across the plasma membrane [5]. A decrease in Na^+^/K^+^-ATPase activity has been implicated in the manifestation of manic-like behaviors in a mouse model [4, 8] that mimics human bipolar disorder. Intracerebroventricular (ICV) administration of ouabain (a Na^+^/K^+^-ATPase inhibitor) in rats has been suggested to mimic some symptoms of human bipolar mania and thus has been used to develop a validated mouse model of manic-like [4–6]. Therefore, we supposed that ouabain treatment may be an appropriate model to investigate the underlying molecular mechanisms between neuroinflammation and bipolar disorder.

CCAAT/Enhancer-Binding Protein Delta (CEBPD) is an inflammatory transcription factor associated with the progression of neurological diseases, including neurodegenerative diseases and central nervous system injuries [3, 9–11]. Previous studies have shown that CEBPD contributes to neuroinflammation, gliosis, and oxidative stress in central nervous system diseases/injuries [3, 9]. However, it remains unclear whether the underlying molecular mechanism that accelerates neuroinflammation through CEBPD activation contributes to the development of psychiatric disorders, particularly bipolar mania.

Matrix metallopeptidase 8 (MMP8), also known as neutrophil collagenase, is involved in neuroinflammation, particularly in the activation of microglia and astrocytes [12]. Previous studies have shown that MMP8 inhibition can decrease the production of reactive oxygen species (ROS) and expression of inflammatory genes in conditions such as Parkinson’s disease and cerebral ischemia [13, 14], thereby reducing neuroinflammation. Furthermore, when astrocytes are stimulated by lipoteichoic acid (LTA), it leads to their activation and an increase in the expression of iNOS, NF-κB, and MAP Kinase. MMP8 inhibitor (M8I) has been shown to reduce the expression of these molecules in astrocytes treated with LTA [12, 13]. However, the molecular mechanism underlying MMP8 expression in astrocytes and its contribution to manic-like behavior remains unknown.

Here, our studies indicate that ouabain treatment upregulates CEBPD expression in astrocytes, consequently inducing the transcription of MMP8. Additionally, we show that the inhibition of MMP8 by M8I can diminish manic-like behavior. This suggests that MMP8 inhibition effectively alleviates manic-like behavior by reducing neuroinflammation through modulation of the astrocytic CEBPD pathway.

## Materials and methods

### Animals

All animal procedures were conducted in strict accordance with the principles outlined in the China Medical University Guide for the Care and Use of Laboratory Animals. Male C57BL/6JNarl mice, aged five weeks, were obtained from the National Applied Research Laboratories, National Laboratory Animal Center, Taiwan. They were housed in a controlled environment, maintaining a temperature of 22 ± 0.5 °C, constant humidity at 60 ± 15%, and a 12-h light–dark cycle, with access to food and water ad libitum.

### Surgery and animal treatment

At six weeks of age, the mice were anesthetized using 2% isoflurane in oxygen and placed on a heating pad. After securing them in a stereotaxic frame, we administered 0.625 nmol of ouabain (Merck-Millipore, Darmstadt, Germany) in 2.0 μL phosphate-buffered saline (PBS) at a rate of 0.2 μL/min [5]. The stereotaxic coordinates for the injection were: 1.25 mm lateral to the midline, 0.55 mm posterior to bregma, and 2 mm ventral to bregma. An equal volume of PBS was injected as the control. M8I was dissolved in 10% DMSO solution in PBS at 5 mg/kg and administered via intraperitoneal injections thrice a week until the seventh day. For the control group, an equivalent volume of 10% DMSO solution in PBS was administered via intraperitoneal injections.

### Cell culture and plasmid transfection

Human primary astrocytes were obtained from ScienCell™ Research Laboratories (Catalog#1800). The cells were seeded in a poly-L-lysine-coated flask and cultured in astrocyte medium (Catalog #1801) containing 10 mL fetal bovine serum (FBS, Cat. No. 0010) and 5 mL astrocyte growth supplement (AGS, Cat. No. 1852). For transfection of astrocytes, the PolyJet reagent (SignaGen Laboratories, SL100688) and plasmids were incubated in a serum-free DMEM medium for 20 min at 23 °C. Subsequently, the transfection reagents were added to the culture dish and incubated at 37 °C in a 5% CO_2_ incubator (Thermo Fisher Scientific) for 24 h.

### Immunofluorescence assay

Frozen sections of whole brain (Control, ouabain, and ouabain + M8I groups) were mounted onto coated glass slides and the OCT embedding medium was dissolved in PBS at 23 °C. Subsequently, the sections were retrieved for 10 min at 90 °C and further blocked with a solution containing 10% normal donkey serum in PBS with 0.1% Tween 20 for 1 h at 23 °C. The sections were then incubated overnight at 4 °C with antibodies against GFAP (Abcam), CEBPD (Abcam), nitrotyrosine (Santa cruz), and Iba1 (Wako) in PBS. The slides were then washed thrice with TBST for 10 min each. After washing, sections were incubated with Alexa Fluor 488- or 555-conjugated secondary antibodies in PBS for 1 h. Following another three washes with TBST for 10 min each, the sections were cover-slipped using ProLong Gold antifade reagent with or without 4ʹ,6-diamidino-2-phenylindole (DAPI) at 23 °C for 10 min. The results were visualized using immunofluorescence confocal microscopy.

### Western blot analysis

Equal amounts of protein extracted from the treated cells were combined with 1 × sample buffer and denatured at 95 °C for 10 min. Proteins were subsequently separated by sodium dodecyl sulfate–polyacrylamide gel electrophoresis (SDS-PAGE) using gels of varying percentages and transferred onto a polyvinylidene difluoride (PVDF) membrane. The membranes were blocked with 5% skim milk in 0.1% Tween-20 in TBST for 1 h. The blocked membranes were then incubated overnight with primary antibodies at 4 °C, as indicated, diluted in TBST. Next, the membranes were washed three times with TBST for 10 min each and incubated with secondary antibodies diluted in TBST for 1 h. After three more washes with TBST, the results were captured using an Azure 400 system.

### Quantitative-PCR

RNA was extracted from the treated cells using an RNA extraction kit (RNeasy Mini Kit; Qiagen, Hilden, Germany). The treated cells were lysed using a lysis buffer, and the resulting mixture was supplemented with 70% ethanol. After extraction, the total RNA was collected and reverse-transcribed into cDNA using an RT-qPCR kit (Bio-Rad). The resulting cDNA was then combined with the indicated primers and enzymes using a CFX96 Touch Real-Time PCR Detection System (BioRad). We determined the cycle threshold (CT) values and calculated the relative expression of the target genes using the 2^−∆∆CT^ method. The specific primers used were: CEBPD (F): GCC ATG TAC GAC GAC GAG AG; CEBPD (R): TGT GAT TGC TGT TGA AGA GGT C; MMP8 (F): GGG CTC TGA GTG GCT ATG AT; MMP8 (R): TTC CTG GAA AGG CAC CTG AT; α-Tubulin (F): CGG GCA GTG TTT GTA GAC TTG G; α-Tubulin (R): CTC CTT GCC AAT GGT GTA GTG C.

### RNA-sequencing (RNA-Seq)

Human primary astrocytes were treated with ouabain, and HA or HA-CEBPD was expressed. Subsequently, RNA was extracted from these cells and transcriptome sequencing was performed using the Illumina NovaSeq 6000 platform.

### Luciferase reporter assay

The pcDNA3-HA/CEBPD construct was prepared, as previously reported [15]. Various promoter regions of the human MMP8 gene were synthesized as DNA fragments and subsequently cloned into the pGL-3 basic vector. Specifically, the following two fragments were synthesized and cloned: MMP8/FL-pGL3 containing sequences from − 817 to + 1, and MMP8/PI-pGL3 containing sequences from -330 to + 1. After co-transfection, luciferase activity in the cell lysates was quantified according to the manufacturer’s instructions for the luciferase assay kit (Promega, E1500). The overexpression of HA-CEBPD was confirmed using Western blot analysis.

### Open-field assay

To assess locomotor activity and the level of anxiety, we conducted the open field test using the protocol modified from the previous studies [16, 17]. Locomotion was evaluated 7 days after ICV and M8I administrations. Mice were habituated in the testing room with 730 lux for 30 min. After habituation, the animals were placed in the center of a white acrylic open box (40 × 40 × 40 cm). The inner area was defined as the 20 × 20 cm area at the center. The activity was recorded and analyzed for 15 min with the Any-Maze automated animal tracking systems.

### Tail-suspension assay

Mice undergoing the tail-suspension assay were acclimated to the testing room, remaining there for 1 h before the actual test. The duration of habituation was uniform for all mice subjected to testing. The tail-suspension assay was performed as previously described with minor modification [18]. In summary, each mouse’s tail was affixed to the edge of a table suspended 30 cm above the ground, and the mobility of the mouse was observed by the observer for a duration of 3 min.

### Statistical analysis

Data were collected from a minimum of three independent cell culture experiments and at least four mice for animal behavioral studies. Statistical significance was determined using the Prism software (version 9.4.0). Data were obtained from replicate experiments and presented as mean ± SEM. Statistical analysis involved the use of unpaired Student’s t-test and one-way analysis of variance (ANOVA), followed by appropriate multiple comparison tests. The significance levels were as follows: *p < 0.05, **p < 0.01, ***p < 0.001, and ****p < 0.0001.

## Results

### CEBPD/Cebpd was highly expressed in astrocytes after ouabain treatment

**Fig. 1:**
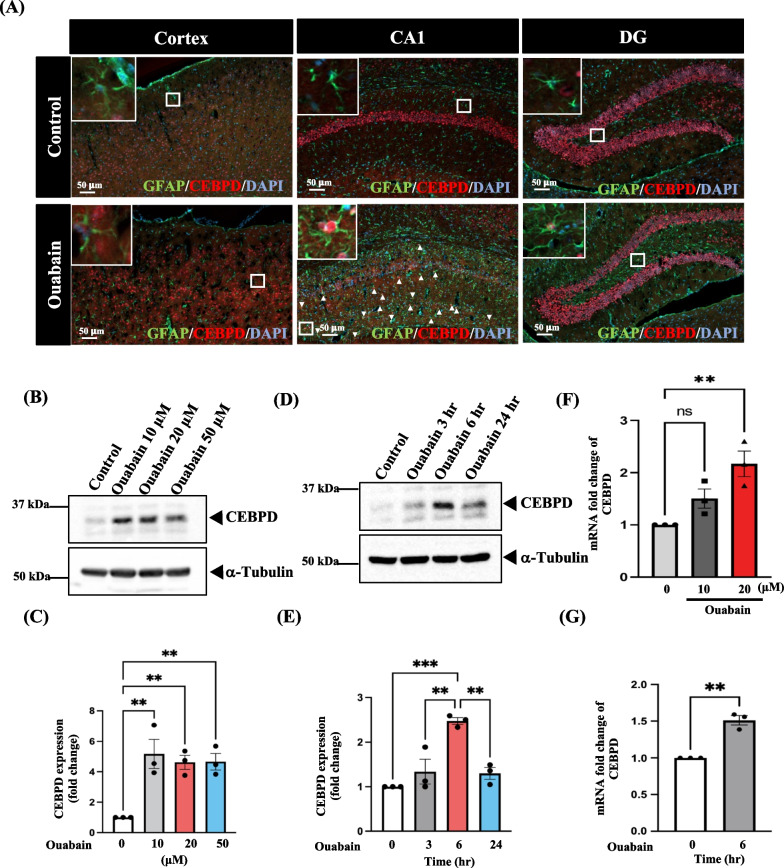
Cebpd/CEBPD is highly expressed in both mouse and human primary astrocytes after ouabain treatment. **A** CEBPD is highly expressed in the astrocytes of CA1 region in ouabain-treated mice. Immunofluorescence assays revealed that CEBPD (red) is predominantly expressed in astrocytes stained with GFAP (green) in mice subjected to ouabain treatment. **B** Western blot analysis showed an increase in CEBPD expression in human primary astrocytes treated with various doses of ouabain. **C** Quantitative data from (**B**) revealed an enhanced intensity of CEBPD in human primary astrocytes. The CEBPD was normalized using α-Tubulin. The quantitative data is presented as means ± SEM; N = 3; analyzed using one-way ANOVA followed by Tukey’s multiple comparisons test, **p < 0.01. **D** Time-dependent treatment of astrocytes with 20 μM ouabain demonstrated an increase in CEBPD expression. **E** Quantitative data from (**D**) displayed the expression levels of CEBPD in astrocytes. The CEBPD was normalized using α-Tubulin. **F** Dose-dependent and **G** time-dependent treatments of astrocytes with ouabain revealed an increase in CEBPD transcription. Q-PCR was conducted to measure CEBPD mRNA levels. Quantitative data are presented as means ± SEM; N = 3; **p < 0.01

Ouabain has reportedly been used to induce manic-like behavior in mice, which exhibit symptoms such as hyperactivity [5, 6]. Neuroinflammation is also associated with the development of manic-like symptoms [19]. CEBPD is an inflammatory transcription factor that contributes to the progression of neurological diseases [3, 10]. Immunofluorescence assay showed that Cebpd was primarily expressed in astrocytes in the hippocampal CA1 region (Fig. 1A). Therefore, we investigated the effect of astrocytic CEBPD on neuroinflammation and the underlying molecular mechanisms in an ouabain-induced manic-like behavior model.

In the cellular model, we used human primary astrocytes to assess whether ouabain could regulate the expression and transcription of CEBPD. We found that CEBPD was upregulated by ouabain treatment in a dose- and time-dependent manner (Fig. 1B–E). Furthermore, the transcription levels of CEBPD also increased after ouabain treatment (Fig. 1F and G). These findings imply a correlation between the ouabain-induced manic-like behavior and heightened CEBPD expression.

### Transcriptome analysis of CEBPD overexpression and ouabain treatment in human primary astrocytes

**Fig. 2:**
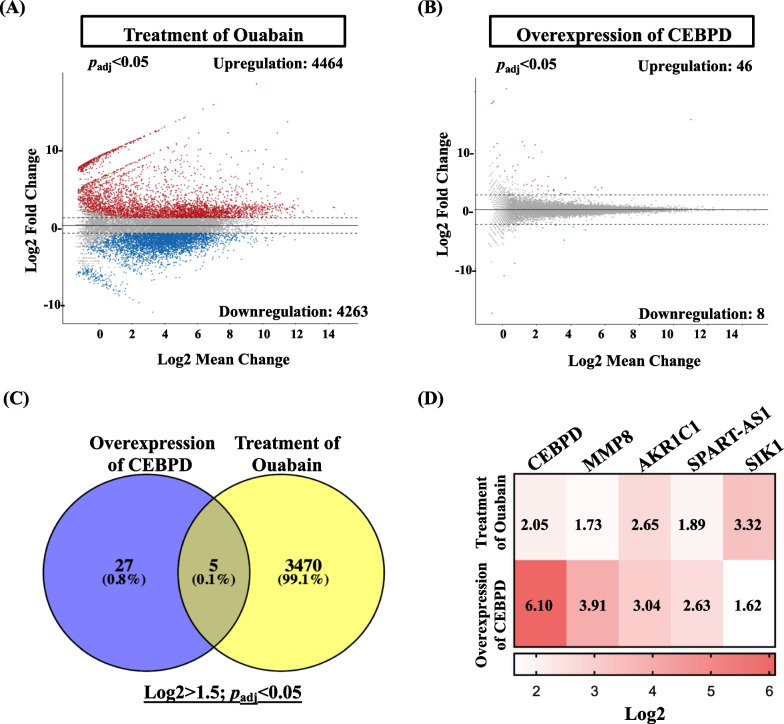
Transcriptome analysis revealed the upregulation of MMP8 in both CEBPD expression and ouabain treatment in astrocytes. **A** Overexpression of CEBPD and **B** treatment with ouabain in human primary astrocytes exhibited changes in the gene expression profile, with profile genes selected based on a significance threshold of *p*adj > 0.05. **C** Venn diagrams and **D** a heatmap illustrated the correlation of the five upregulated genes. The number on the heatmap represents the Log2 expression value. All Next-Generation Sequencing (NGS) analyses were conducted using cells collected from a single experiment to unveil these findings. Subsequently, the analyzed data was validated through a Q-PCR assay to ensure accuracy

RNA-seq analysis was used to examine downstream gene expression. Additionally, we investigated downstream target gene expression following the overexpression of CEBPD and treatment with ouabain in human primary astrocytes. As illustrated in Fig. 2A, 4464 genes were significantly induced in human primary astrocytes, whereas 4263 genes were inhibited by ouabain treatment. In addition, 46 genes were upregulated and eight were downregulated following overexpression of CEBPD in human primary astrocytes (Fig. 2B). Venn diagrams and heat map analyses revealed that five genes were correlated in the upregulated dataset (Fig. 2C and D). Among these, MMP8 exhibited a significant increase, and its promotion of neuroinflammation has been reported. Subsequently, we investigated the function of MMP8, a downstream target gene of CEBPD, in ouabain-induced manic-like behavior.

### CEBPD regulated MMP8 transcription through promoter regulation in human primary astrocytes

**Fig. 3:**
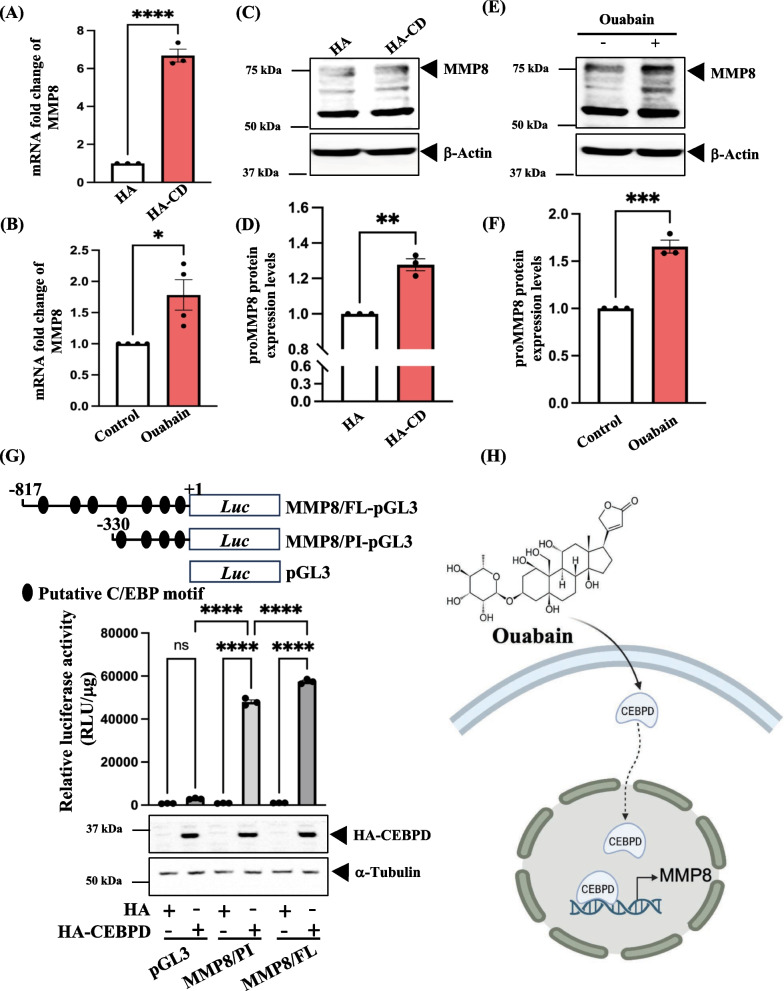
CEBPD regulates MMP8 transcription in human primary astrocytes through the MMP8 promoter region. MMP8 transcription was increased in (**A**) CEBPD overexpression or (**B**) ouabain treatment in astrocytes. Q-PCR analyses were performed using specific primers with cDNA harvested from either CEBPD overexpression or 20 μM ouabain treatment in astrocytes. Quantitative data is presented as means ± SEM; N = 3 (**A**) or N = 4 (**B**); analyzed using a two-tailed unpaired Student’s t-test, *p < 0.05 and ****p < 0.0001. The protein expression of MMP8 was also increased in (**C**) CEBPD overexpression or (**E**) ouabain treatment in astrocytes. Quantitative data from (**D**) and (**F**) depict the expression levels of CEBPD in astrocytes. The CEBPD was normalized using β-Actin. Quantitative data is presented as means ± SEM; N = 3; analyzed using a two-tailed unpaired Student’s t-test, **p < 0.01 and ***p < 0.001. **G** Identification of CEBPD binding motifs on the MMP8 promoter region involved co-transfecting the MMP8 promoter reporter vectors with either the HA or HA-CEBPD expression vectors into human primary astrocytes. After 24 h, cell lysates were harvested for Western blot analysis and luciferase assays. **H** The schematic illustrates CEBPD-regulated MMP8 expression in astrocytes

Based on the aforementioned results, overexpression of CEBPD or treatment with ouabain resulted in the upregulation of MMP8 in human primary astrocytes. To validate our transcriptome data, we assessed MMP8 transcription in both CEBPD overexpression and ouabain treatment conditions. The data confirmed that both CEBPD overexpression and ouabain treatment upregulated MMP8 transcription (Fig. 3A and B). Furthermore, the protein expression of MMP8 was also increased in human primary astrocytes subjected to CEBPD overexpression or ouabain treatment (Fig. 3C–F). Using a luciferase reporter assay, we identified a potent CEBPD-responsive region in the MMP8 promoter at − 330/ + 1 bp (Fig. 3G). These findings strongly suggest that CEBPD regulates MMP8 expression by binding to the promoter region of MMP8 (Fig. 3H).

### M8I, an inhibitor of MMP8, effectively reduced ouabain-induced manic-like behavior in stressed mice

**Fig. 4:**
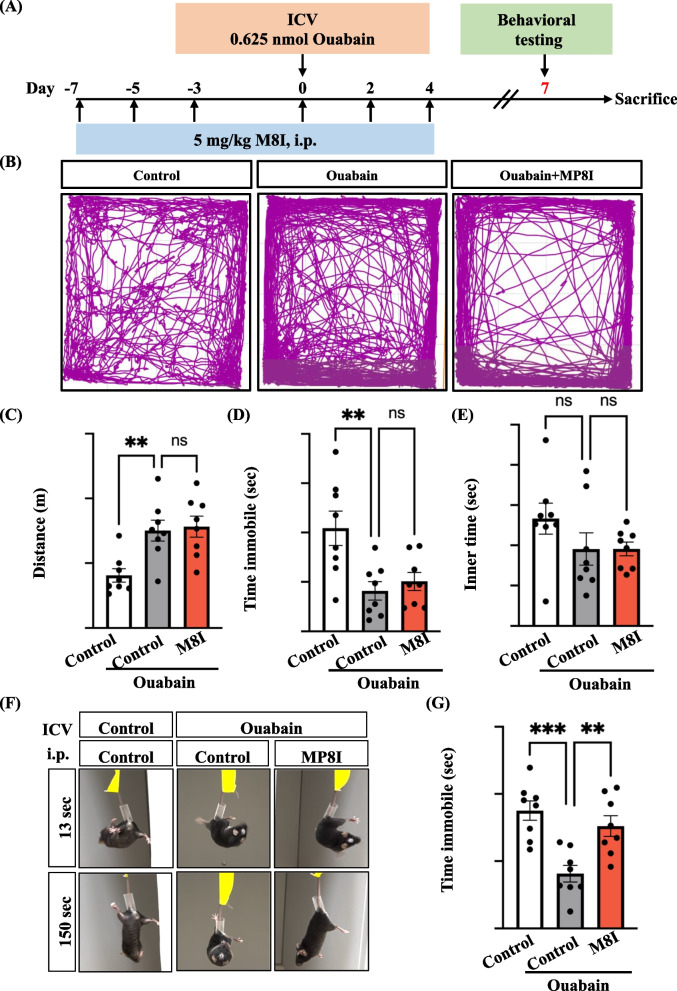
M8I, an MMP8 inhibitor, attenuates ouabain-induced manic-like behavior in mice. **A** This illustration outlines the timeline for the experimental procedures. **B** Representative images depict the exploratory behavior of the mouse in the open field. **C** The distance traveled in the open field reflects the mice’s movement. **D** The time spent immobile indicates periods when the mice were not moving. **E** The time spent in the inner area of the open field test measures anxiety-related behavior. **F** Representative images show extreme behavior testing in tail-suspension assay. **G** Quantitative data from (**F**) reveals a decrease in immobility time in ouabain-injected mice compared to control or M8I-treated mice. These tests were conducted on day 7 after ouabain was injected into mice via ICV injection. Intraperitoneal injection of M8I (5 mg/kg) began seven days before ouabain injection and continued until the open field test and tail-suspension assay. Quantitative data is presented as means ± SEM; N = 8; analyzed using two-way ANOVA followed by Tukey’s multiple comparisons test, **p < 0.01 and ***p < 0.001

It has been reported that MMP8 gets activated in astrocytes and microglia, contributing to the progression of neuroinflammation, which includes heightened levels of inflammatory cytokines and oxidative stress [13, 14]. Our results also demonstrated that the expression of MMP8 increased with CEBPD overexpression and treatment with ouabain in primary human astrocytes (Fig. 3). In this study, we utilized ouabain-treated mice to evaluate manic-like behavior using open-field and tail-suspension assays. Initially, mice were pretreated with M8I for seven days, followed by a single ICV injection of ouabain. Subsequently, we continued the injection of M8I for seven days, as depicted in Fig. 4A. In Fig. 4B–D, it can be seen that both treated and untreated ouabain-injected mice exhibited higher levels of manic-like behavior in comparison to the PBS control mice in the open-field assay, which is an unmotivated condition. Additionally, we assessed the time spent in the center by all three groups of mice and found no significant differences (Fig. 4E). Furthermore, we conducted an extreme behavior test using a tail-suspension assay to evaluate the depressive abilities of mice under extreme conditions. We observed a significant reduction in immobility time in ouabain-injected mice, whereas treatment with M8I reversed this effect, similar to that in the control groups (Fig. 4F and G). Our findings suggest that M8I can mitigate manic behavior in ouabain-injected mice under stressful conditions.

### M8I reduced the activation of astrocytes and microglia in ouabain-treated mice

**Fig. 5:**
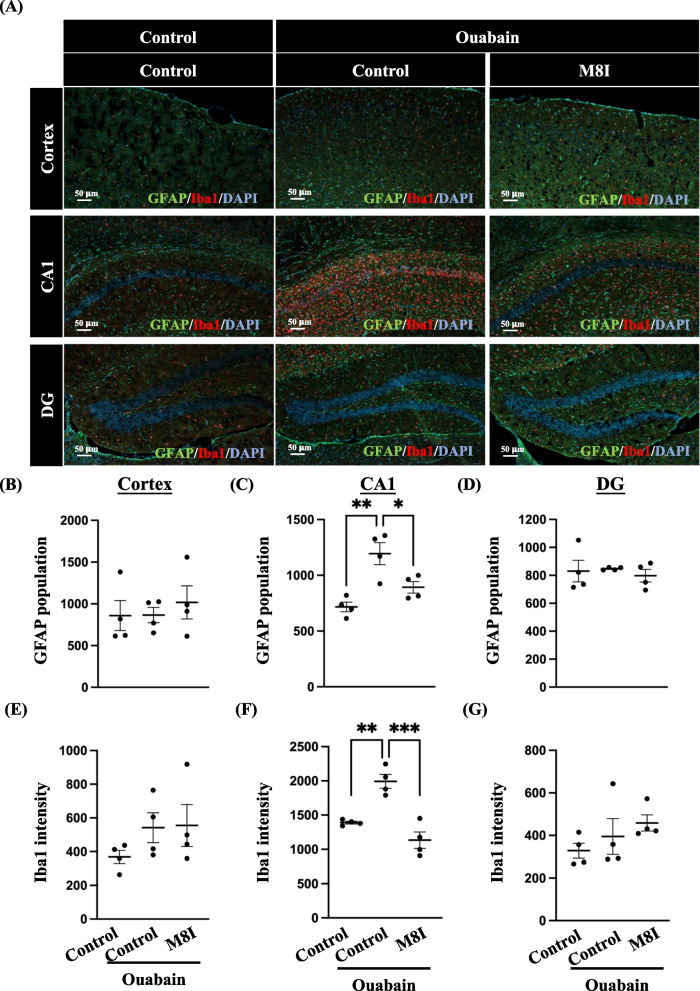
M8I treatment reduces glial cell activation in ouabain-injected mice. **A** The activation of astrocytes and microglia was assessed using immunofluorescence. Brain sections were collected from three groups of mice and subjected to immunofluorescence staining using GFAP (astrocyte marker, green) and Iba1 (microglia marker, red). The quantitative analysis of astrocyte populations in **B** the cortex, **C** the CA1 region, and **D** DG revealed an increase in the CA1 region in ouabain-injected mice, while M8I treatment reduced the astrocyte population. Similarly, the quantitative analysis of Iba1 intensity is depicted in **E** the cortex, **F** the CA1 region, and **G** the DG. M8I treatment, furthermore, resulted in a reduction of the Iba1 intensity, specifically in the CA1 region. Cell numbers and intensities were quantified by the QuPath software Cell Detection function. Quantitative data is presented as means ± SEM; N = 4; analyzed using one-way ANOVA followed by Tukey’s multiple comparisons test, *p < 0.05, **p < 0.01, and ***p < 0.001

M8I has been reported to reduce glial cell activation in Parkinson’s disease [13]. However, it remains unclear whether M8I inhibits glial cell activation in ouabain-induced manic-like behavior. Immunofluorescence analysis revealed increased astrocyte activation in the hippocampal CA1 region of the ouabain-injected mice. Conversely, treatment with M8I reduced astrocyte activation in ouabain-injected mice (Fig. 5A–D). Similarly, microglial activation was significantly increased in the hippocampal CA1 region of ouabain-injected mice; however, treatment with M8I effectively inhibited microglial activation (Fig. 5A, E–G). These findings suggest that the downstream target gene MMP8, which is regulated by astrocytic CEBPD, plays a crucial role in ouabain-induced glial activation.

### M8I attenuated the expression of oxidative stress marker nitrotyrosine in astrocytes of ouabain-treated mice

**Fig. 6:**
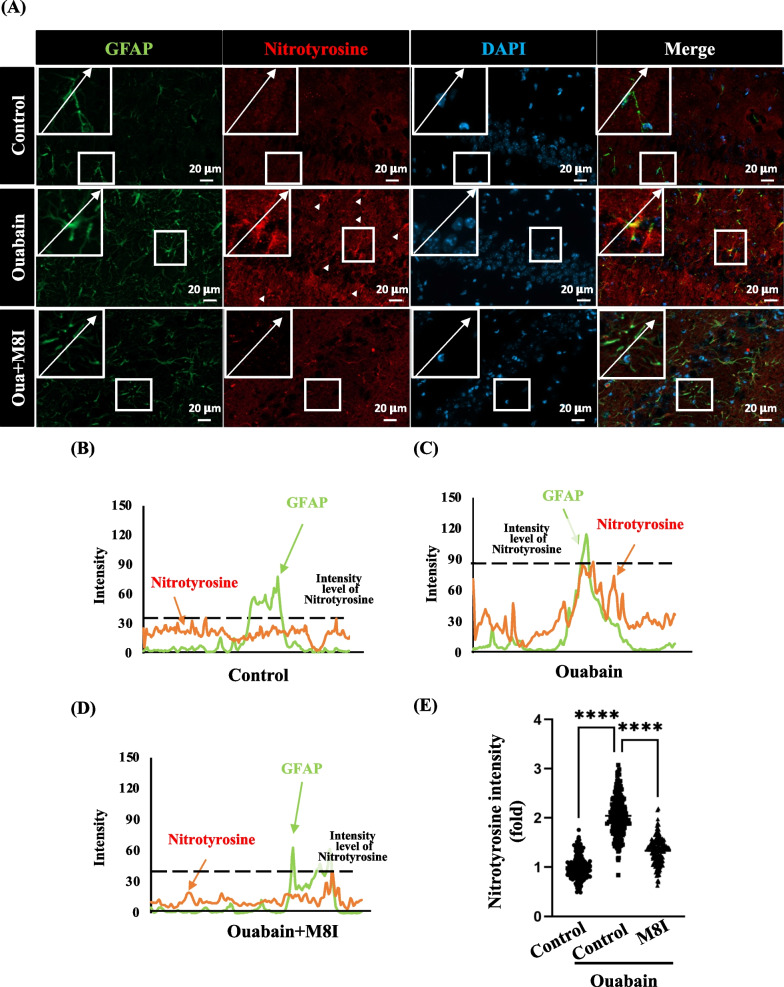
M8I treatment reduces oxidative stress in astrocytes in ouabain-injected mice. **A** The expression of nitrotyrosine in astrocytes was assessed using immunofluorescence. Brain sections were collected from three groups of mice and subjected to immunofluorescence staining with GFAP (astrocyte marker, green) and nitrotyrosine (oxidative stress marker, red). **B**–**D** Tracking (arrows shown in A) of the intensities for nitrotyrosine (red) and GFAP (green) in the brain sections indicated the intensity of nitrotyrosine produced in astrocytes. The analysis of nitrotyrosine intensity revealed an increase in the ouabain-injected mice groups compared to the control or M8I-treated groups. **E** The quantitative analysis of nitrotyrosine intensity has revealed an increase in the CA1 region of ouabain-injected mice, while M8I treatment reduced the nitrotyrosine intensity in the CA1 region of ouabain-injected mice. Cell intensities were quantified by the QuPath software Cell Detection function. Quantitative data is presented as means ± SEM; and analyzed using one-way ANOVA followed by Tukey’s multiple comparisons test, ****p < 0.0001. The cell number counter utilizes Qupath, and the cell counts are as follows: control (182 cells), ouabain (214 cells), and ouabain + M8I (162 cells)

To investigate the role of MMP8 in oxidative stress, we conducted immunostaining for nitrotyrosine, an oxidative stress marker [3, 20], in brain sections from the three different groups of mice. Our findings indicate an increased intensity of nitrotyrosine production in astrocytes with the presence of GFAP in the brain sections (Fig. 6A–C, E). However, this intensity markedly decreased in astrocytes following the administration of M8I in ouabain-injected mice (Fig. 6A, D, E). These results strongly support the notion that MMP8 contributes to oxidative stress in ouabain-treated mice, whereas M8I (an inhibitor of MMP8) effectively mitigates oxidative stress in the astrocytes of ouabain-treated mice.

## Discussion

Recent studies have highlighted the correlation between neuroinflammation and the pathological progression of psychiatric disorders [6, 21]. Specifically, current research indicates that mood fluctuations in bipolar disorder are associated with changes in the inflammatory status [21]. Furthermore, elevated levels of inflammatory cytokines and oxidative stress have been shown to correlate with the expression of CEBPD, particularly in astrocytes [3, 22]. In our study, we observed CEBPD expression in astrocytes of ouabain-injected mice. Moreover, we also demonstrated that ouabain treatment of human primary astrocytes activated CEBPD and triggered the downstream expression of MMP8. Importantly, administration of M8I, an MMP8 inhibitor, effectively reversed ouabain-induced manic-like behavior, alleviated glial activation, and reduced oxidative stress. These findings underscore the significance of our study and highlight that CEBPD may play a significant role in neurodegenerative and psychiatric diseases.

CEBPD has been reported to contribute to the progression of neurodegenerative diseases and central nervous system injuries by promoting astrogliosis, generating reactive oxygen species (ROS), recruiting microglia into lesions, and inhibiting macrophage-mediated phagocytosis [3, 9, 23, 24]. CEBPD can be activated by IL-1β, TNF-α, and β-amyloid [3, 24]. However, it remains unclear whether CEBPD contributes to the development of psychiatric disorders, specifically the manifestation of manic-like symptoms in bipolar disorder. Na^+^/K^+^-ATPase is expressed in all brain cells, including astrocytes [25, 26] and neuronal cells [27, 28]. In our study, we observed that ouabain, an inhibitor of Na^+^/K^+^-ATPase, activated CEBPD expression in astrocytes and induced manic-like behavior. This study represents the initial investigation into the role of CEBPD in the development of manic-like symptoms in bipolar disorder, particularly its impact on manic behavior.

Lithium has an important role in the treatment of mood episodes or the maintenance phase of treatment of bipolar disorder [29]. The activation of GSK3β has been reported in active astrocytes [23, 30], and it is known that Lithium Chloride acts as an inhibitor of GSK3β, which can inhibit the phosphorylation of CEBPD [23]. CEBPD phosphorylated at Ser167 in astrocytes reportedly stimulates microglial activation and migration by activating MMP1, MMP3, and monocyte chemotactic protein-1 (MCL-1) [23]. However, it remains unclear whether phosphorylated CEBPD is activated in ouabain-treated astrocytes or ouabain-injected mice. In particular, our results showed microglial activation in ouabain-injected mice. Consequently, we hypothesized that microglial activation might be mediated by the activation of astrocytic CEBPD, specifically by CEBPD phosphorylation.

Nucleocytoplasmic transport plays a vital role in facilitating the nuclear translocation of transcription factors that influence gene transcription. Moreover, defects in nucleocytoplasmic transport have been associated with various neurological diseases, including ALS and dementia [31–33]. The nuclear pore complex is composed of approximately thirty nucleoporins that collectively form a crucial gateway [31, 33]. However, it remains unclear whether nucleocytoplasmic transport plays a role in the development of psychiatric disorders, specifically contributing to manic-like symptoms in bipolar disorder. The immunofluorescence data in Fig. 1A clearly demonstrates that astrocytic CEBPD is predominantly localized in the nucleus. Therefore, we hypothesize that enhanced activation of nucleocytoplasmic transport or inflammatory transcription in ouabain-treated mice serves additional functions to facilitate passage through the nuclear pore complex. However, we must acknowledge the potential involvement of CEBPD expression and functionality in neuron cells, prompting further investigation into the reasons behind the observed decrease in expression levels within the CA1 region of ouabain-treated mice (Fig. 1A). This question remains an open avenue for future exploration, focusing on two key aspects: (1) Delving into the mechanism by which ouabain, a Na + /K + -ATPase inhibitor, diminishes CEBPD expression in neuron cells. (2) Unraveling the intricate details of the differential expression patterns of CEBPD in astrocytes and neuron cells.

The MMP family plays a significant role in various neurological diseases and injuries, including Alzheimer’s disease, Amyotrophic lateral sclerosis (ALS), and spinal cord injuries [10, 23, 34, 35]. CEBPD has been identified as a regulator of MMP1, MMP3, and MMP9 in the gene profiles of cancer cells [23, 36]. Our data demonstrates a significant increase in MMP8 levels in human primary astrocytes. Predictive promoter analysis revealed that MMP8 contains multiple CEBPD-binding motifs. Our results indicated that CEBPD regulates MMP8 expression through promoter regulation. Previous studies have shown that treatment with MMP8 inhibitors can reduce LTA-induced inflammation and oxidative stress in astrocytes. MMP8 inhibitors can also mitigate neuroinflammation in a Parkinson’s disease model with LRRK2 G2019S mutation [12, 13, 37]. However, the underlying molecular mechanism of astrocytic CEBPD/MMP8 axis regulation through Na^+^/K^+^-ATPase in ouabain-treated mice remains a subject for future investigation.

Open-field and tail-suspension assays are commonly used to assess behaviors associated with mania or depression [5, 6]. The open-field assay primarily evaluates manic-like behavior related to unmotivated conditions. In contrast, the tail suspension assay, which is similar to the forced swimming assay, has been designed to measure depressive-like behavior under stressful conditions. Previously, a forced swimming assay showed that mice treated with ouabain exhibited decreased immobility time compared to control mice at seven days [6], which is indicative of extremely manic-like behavior. Similarly, our results demonstrated a reduction in immobility time in ouabain-treated mice compared to control mice when using the tail-suspension assay. Notably, treatment with M8I in ouabain-treated manic-like mice revealed a significant difference in the tail-suspension assay, which evaluates behavior under stressful conditions, but not in the open-field assay, which assesses behavior under unmotivated conditions. These findings suggest that the treatment with M8I primarily targets manic-like behavior under stressful conditions (Fig. 4).

## Conclusion

**Fig. 7:**
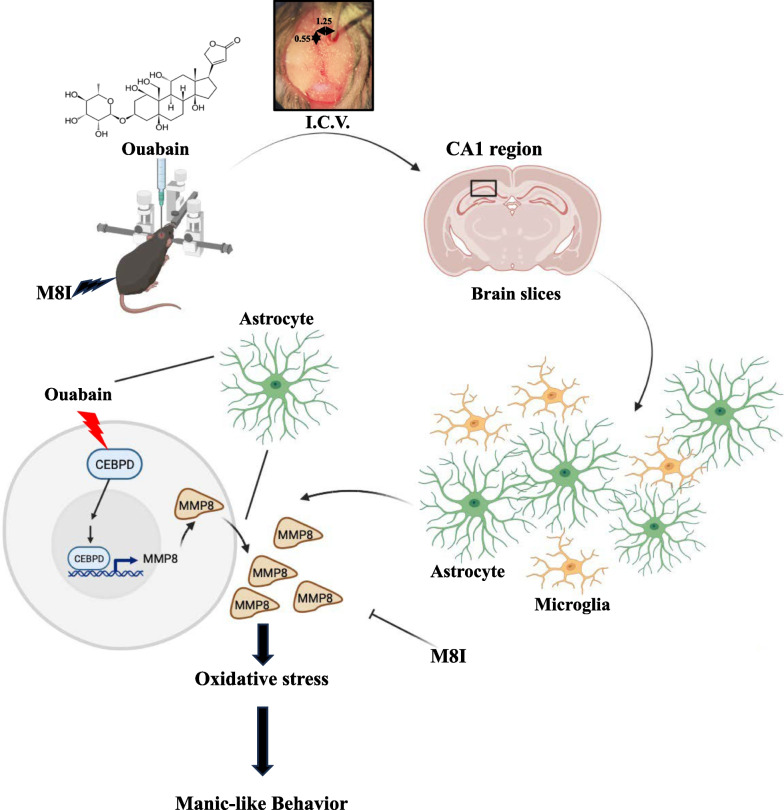
Schematic diagram illustrating the impact of astrocytic CEBPD/MMP8 axis on oxidative stress in ouabain-induced manic-like mice. Ouabain can induce glial cell activation and enhance the expression of astrocytic CEBPD, particularly in the CA1 region. Subsequently, astrocytic CEBPD can regulate the expression of MMP8, which, in turn, promotes oxidative stress by increasing nitrotyrosine levels. Treatment with M8I (an inhibitor of MMP8) reduces glial cell activation and oxidative stress in ouabain-injected mice, decreasing manic-like behavior. Ultimately, this sequence of events reveals that neuroinflammation is linked to manic-like behavior as occurs in bipolar disorder through the activation of the astrocytic CEBPD/MMP8 pathway

Our study revealed that ouabain activated CEBPD in both mouse and human primary astrocytes. Furthermore, transcriptome analysis showed a significant upregulation of MMP8 in astrocytes overexpressing CEBPD or those treated with ouabain. Our investigation revealed that CEBPD regulated MMP8 transcription by binding to its promoter region. The animal study showed that M8I (an MMP8 inhibitor) effectively reversed ouabain-induced manic-like behavior. Histological analysis further demonstrated that M8I reduced microglial and astrocyte activation, while attenuating oxidative stress in astrocytes. These findings suggest that MMP8 may be a promising therapeutic target, potentially opening new translational avenues for treating manic-like behavior in bipolar disease. Furthermore, this study is the first to identify the novel pathway of the CEBPD/MMP8 axis in astrocytes as a regulator of oxidative stress and neuroinflammation in the ouabain-induced manic-like model (Fig. 7).

## Data Availability

The data supporting this study are available upon reasonable request to the corresponding author.

## Abbreviations

CEBPD: CCAAT/enhancer-binding protein delta
HA-CD: HA tag-CCAAT/enhancer-binding protein delta
MMP: Matrix metallopeptidase
Na^+^/K^+^-ATPase: Sodium–potassium ATPase pump
Oua: Ouabain
M8I: Matrix metallopeptidase 8 inhibitor
NGS: Next generation sequencing
ROS: Reactive oxygen species
LTA: Lipoteichoic acid
DAPI: 4ʹ,6-Diamidino-2-phenylindole
SDS-PAGE: Sodium dodecyl sulfate–polyacrylamide gel electrophoresis
PVDF: Polyvinylidene difluoride
CT: Cycle threshold
CA1: Cornu ammonis 1
DG: Dentate gyrus
GFAP: Glial fibrillary acidic protein
Iba1: Ionized calcium-binding adaptor molecule 1
ICV: Intracerebroventricular injection
i.p. injection: Intraperitoneal injection
MCL-1: Monocyte chemotactic protein-1
LiCl: Lithium chloride
ALS: Amyotrophic lateral sclerosis
